# The DSM-5 diagnosis of nonsuicidal self-injury disorder: a review of the empirical literature

**DOI:** 10.1186/s13034-015-0062-7

**Published:** 2015-09-28

**Authors:** Maria Zetterqvist

**Affiliations:** Department of Clinical and Experimental Medicine, Linköping University, 581 85 Linköping, Sweden; Department of Child and Adolescent Psychiatry, Linköping University, 581 85 Linköping, Sweden

**Keywords:** Nonsuicidal self-injury disorder, Adolescents, DSM-5, Review

## Abstract

With the presentation of nonsuicidal self-injury disorder (NSSID) criteria in the fifth version of the Statistical and Diagnostic Manual of Mental Disorders (DSM-5), empirical studies have emerged where the criteria have been operationalized on samples of children, adolescents and young adults. Since NSSID is a condition in need of further study, empirical data are crucial at this stage in order to gather information on the suggested criteria concerning prevalence rates, characteristics, clinical correlates and potential independence of the disorder. A review was conducted based on published peer-reviewed empirical studies of the DSM-5 NSSID criteria up to May 16, 2015. When the DSM-5 criteria were operationalized on both clinical and community samples, a sample of individuals was identified that had more general psychopathology and impairment than clinical controls as well as those with NSSI not meeting criteria for NSSID. Across all studies interpersonal difficulties or negative state preceding NSSI was highly endorsed by participants, while the distress or impairment criterion tended to have a lower endorsement. Results showed preliminary support for a distinct and independent NSSID diagnosis, but additional empirical data are needed with direct and structured assessment of the final DSM-5 criteria in order to reliably assess and validate a potential diagnosis of NSSID.

## Background

Nonsuicidal self-injury (NSSI), defined as the deliberate, self-inflicted destruction of body tissue without suicidal intent and for purposes not socially sanctioned, includes behaviors such as cutting, burning, biting and scratching skin [1]. NSSI is especially prevalent during adolescence with mean and pooled rates of 17–18% in recent reviews of community samples [2, 3]. In clinical samples of adolescents rates are even higher, with 40% or more reporting NSSI [4]. During the last decades there have been ongoing discussions regarding the conceptualization and diagnostic organization of NSSI. In the diagnostic nomenclature NSSI has been limited to a symptom of borderline personality disorder (BPD), described as suicidal behavior, gestures, threats or self-mutilating behavior [5]. Arguments have been put forward that NSSI should be a separate syndrome [6–11]. In the early 1980s Pattison and Kahan [11] and Kahan and Pattison [9] described the typical patterns of a separate deliberate self-harm syndrome, proposing that it should be included in the fourth version of the Diagnostic and Statistical Manual of Mental Disorders (DSM-IV) [5], with inability to resist the impulse to injure oneself, increased sense of tension prior to the act and experience of release/relief after the act as essential features. Later, Favazza and Rosenthal [6, 7] suggested DSM inclusion of a repetitive self-mutilation syndrome and complemented earlier descriptions by adding preoccupation with harming oneself. In 2005 Muehlenkamp [10] also proposed that self-injurious behavior should be a separate clinical syndrome, emphasizing the absence of conscious suicidal intent, the inability to resist NSSI impulses, the negative affective/cognitive state prior to and the relief after NSSI, as well as the preoccupation with and repetitiveness of the behavior. These earlier features overlap to a large extent with the suggested Shaffer and Jacobson [12] NSSI criteria proposed to the DSM-5 [13] Childhood Disorder and Mood Disorders work group for inclusion as a DSM-5 disorder, in that they describe the functional, motivational and emotional aspects of NSSI [14]. The criteria have been revised several times during the work progress, mainly concerning their organization [12, 13, 15].

Shaffer and Jacobson [12] pinpointed several reasons in their rationale for reclassifying NSSI: NSSI is associated with clinical and functional impairment; the classification of NSSI solely as a symptom of BPD is inconsistent with recent evidence; NSSI needs to be separated from suicide attempts; studying NSSI purely within a BPD context or as a manifestation of suicidality will hamper research and treatment of NSSI; a standardized definition of clinically significant NSSI would facilitate comparisons of findings from different studies and improve communication and clarity in clinical care.

There is general consensus that there is an association between BPD and NSSI [16–19], but that NSSI is not unique to BPD. NSSI is also associated with other personality disorders [19, 20] and to several axis I symptomatologies [16, 19–21], and may also be present without any psychiatric comorbidities [22]. To classify NSSI purely as a criterion of BPD implies that it does not have clinical significance outside the BPD context [23].

Furthermore, not separating suicidal behaviors and NSSI can lead to inaccurate case conceptualization, risk assessment, treatment and iatrogenic hospitalization [23]. Empirical differences have been found between adolescents engaging in different kinds of self-injurious behaviors with and without suicidal intent (e.g., [18]). Ignoring intent in describing self-injury can lead to an overestimation of the prevalence of suicide attempts and prevent correct identification of specific risk factors for the respective behaviors [24]. The relationship between NSSI and suicide attempts is complex and nuanced [25] and there is general agreement that there is an overlap between nonsuicidal and suicidal self-injury [20, 26]. Recent longitudinal research has found that NSSI predicts suicide attempts in adolescents [27–29] and that the high co-occurrence between the two can be understood in the light of NSSI increasing the risk for suicidal behavior [30]. Arguments have thus been put forward that nonsuicidal and suicidal self-injury need to be differentiated on the basis of differences in intent, lethality, methods, prevalence, frequency and functions [10, 31]. It has also been argued that new definitions of NSSI disorder and suicidal behavior disorder would facilitate comparisons between studies [32].

Despite the fact that NSSI is prevalent and impairing in adolescents, it has not been given any psychopathological significance except as a symptom of BPD until DSM-5 [22]. Improved communication, more precise definition and clearer implications for prognosis and treatment are thus advocated [22, 33], allowing NSSI to be highlighted and treated outside the BPD context [22, 34, 35]. However, doubts have also been voiced [36], mainly concerning the issue of suicidal intent and how the relationship between NSSI and suicidal behaviors should be conceptualized. Critics argue that suicidal or nonsuicidal intent is wrongly reduced to a dichotomy, instead of being conceptualized as a multidimensional construct where the ambiguity and the difficulty in arriving at a valid and reliable assessment of intent need to be acknowledged. Critics further claim that the term nonsuicidal is questionable due to the afore-mentioned overlap between suicidal thoughts and behaviors and NSSI. There is also concern that a diagnosis could increase stigmatization in a young age group and that the lack of empirical support for an NSSI diagnosis argues for caution at this stage [37, 38].

Due to the novelty of the suggested NSSI criteria, crucial empirical data have only recently begun to emerge [39]. The NSSI criteria were finally placed in Section III of DSM-5: Emerging Measures and Models, as a condition that requires further study [13], due to lack of reliability in the clinical trial. Two of the child/adolescent sites had inadequate sample sizes, which were insufficient to obtain accurate estimates of kappa. The third field trial was successful, but the test–retest reliability was unacceptable [40, 41]. Since empirical data are crucial at this point of the diagnostic process, this paper aims at reviewing the empirical literature on the NSSI disorder (NSSID) diagnosis up to the present time.

## Method

Electronic searches were made using the scholarly database search engines Pubmed, PsycInfo, Scopus and Academic Search Premier up to May 16, 2015. The following search terms were used: “non-suicidal self-injury” AND “dsm”; “nonsuicidal self-injury” AND “dsm”; “self-injury” AND “dsm”; “self-harm” AND “dsm”. Abstracts of identified articles were reviewed for inclusion and exclusion criteria. In addition, reference lists of articles were checked so as not to miss other articles that had not appeared in the electronic search. Articles were included if they were peer-reviewed empirical research of the suggested DSM-5 NSSI criteria on samples with children, adolescents and young adults and were written in English. Since empirical data on the NSSI diagnosis are only now emerging, the few articles concerning adults only were also included, but presented separately.

## Results

A total of 16 published studies were found that presented empirical data on NSSID. Four studies used the final DSM-5 [13] criteria, while others used some or all of the earlier criteria [12, 15]. Of these, one based the empirical data on clinicians’ ratings [42] and two [43, 44] were new analyses of study populations already included [45, 46]. Ten studies included adolescents [14, 23, 44, 46–52], of which two also included older children [47, 48]. Four studies included young adults [51–54] (only or in addition to adolescents) and three were limited to adults only [43, 45, 55]. See Table 1 for empirical studies.

**Table 1:** Empirical studies of the nonsuicidal self-injury disorder diagnosis

References	Type of sample	Sample size (female %)	Age group Range Mean age (SD)	Country	NSSI criteria used	Prevalence (%)	Female (%) of those with NSSID	Instruments assessing NSSI disorder criteria
Albores-Gallo et al. [47]	School	533 (54)	Children and adolescents 11–17 years 13.37 (0.95)	Mexico	Shaffer and Jacobson^a^ (2009)	5.6	66.7	Self-Injury Questionnaire (self-report)
Andover [55]	Community	548 (46.5)	Adults 18–73 years 35.70 (12.23)	US	APA^b^ (2013)	2.6 (11.2 of those with an NSSI history)	50.0	Questions developed for DSM-5 criteria (self-report)
Barrocas et al. [48]	School	665 (55.0)	Children and adolescents 7–16 years 11.6 (2.4)	US	Shaffer and Jacobson^c^ (2009)	1.5		SITBI interview FASM CDI (self-report)
Bracken-Minor and McDevitt-Murphy [54]	College	480 (79.8)	Young adults 18–54 years 21.30 (5.69)	US	Shaffer and Jacobson^d^ (2009)	12.9^C^		ISAS (self-report)
Fischer et al. [49]	Clinical inpatient	111 (65.8)	Adolescents 12–19 years 15.38 (1.72)	Germany	APA (2013)	36.9		SITBI interview
Glenn and Klonsky [23]	Clinical inpatient and partial hospitalization	198 (74)	Adolescents 12–18 years 15.13 (1.38)	US	Shaffer and Jacobson^e^ (2009)	50 (78 of self-injuring sample)	86.7	ISAS (self-report)
Gratz et al. [53]	Community	107 with NSSI (80)	Young adults 18–35 years 23.86 (4.87)	Canada and US	APA (2013)	37 of repetitive NSSI sample	85.0	CANDI Structured diagnostic interview
In-Albon et al. [50]	Clinical inpatient	73 (100)	Adolescents 13–18 years	Germany and Switzerland	APA (2012)	56.2	Only female sample	Clinical interview DSM-5 criteria reformulated questions
Manca et al. [14]	School	953	Adolescents	Italy	Shaffer and Jacobson^f^ (2009)	3.1 (49.2 of repetitive NSSI sample)		DSHI R-NSSI-Q SBQ-R (self-report)
Odelius and Ramklint [51]	Clinical outpatient	39 (87.2)	Adolescents and young adults 13–25 years 21 (1.9)	Sweden	Shaffer and Jacobson (2009)	46.2 of NSSI sample	83.3	Clinical interview DSM-5 criteria reformulated questions
Selby et al. [45]^A^	Clinical outpatient	571 (53)	Adults	US	Shaffer and Jacobson^g^ (2009)	11.4	50.8	Chart data
Washburn et al. [52]	Clinical inpatient, partial hospitalization and intensive outpatient	511 (90.0)	Adolescents and young adults 12–52 years 17.3 (6.2)	US	APA (2013)	74.0 of NSSI sample		ABASI (self-report)
Zetterqvist et al. [46]^B^	School	3,060	Adolescents 15–17 years 16.4 (0.89)	Sweden	APA^h^ (2012)	6.7 (18.8 of NSSI sample)	83.4	SITBI-SF-SR FASM (self-report)

An additional study by Lengel and Mullins-Sweatt [42] was identified and is referred to in the text, but its focus is on clinicians’ assessment of criteria.

*ABASI* Alexian Brothers Assessment of Self-Injury, *DSHI* Deliberate Self-Harm Inventory, *NSSI* nonsuicidal self-injury, *ISAS* Inventory of Statements about Self-Injury, *FASM* Functional Assessment of Self-Mutilation, *SBQ-R* Suicide Behaviors Questionnaire—Revised, *SITBI-SF-SR* Self-Injurious Thoughts and Behaviors Interview Short-Form Self-Report, *CDI* Children’s Depression Inventory, *CANDI* Clinician Administered Nonsuicidal Self-Injury Disorder Index, *R-NSSI-Q* Repetitive Non-Suicidal Self-Injury Questionnaire.

^a^Criterion D not assessed.

^b^Criteria D and F not assessed.

^c^Criteria B1, B2, B3 and D not assessed.

^d^Criteria B2, B3, C and D not assessed.

^e^Criteria B2, B3, D not assessed.

^f^Criterion D not assessed

^g^Criteria B1, B2, B3, B4 and C not assessed.

^h^Criterion D not assessed

^A^Same study sample as in Ward et al. [43].

^B^Same study sample as in Zetterqvist et al. [44].

^C^The title of the study may have led to a bias in participant selection with high rates of NSSI.

### NSSI disorder characteristics

Prevalence of NSSID in child and adolescent community samples ranged from 1.5 to 5.6% [47, 48]. In community samples of adolescents only, 3.1–6.7% met NSSID criteria [14, 46], as compared to 18.8% of those with an NSSI history [46] and 49.2% of those with repetitive NSSI [14]. Equivalent rates in a young adult community sample with repetitive NSSI were 37% [53]. Prevalence in adolescent and young adult clinical samples ranged from 36.9 to 50% [23, 49] while 46.2 to 78% [23, 50–52] of those with an NSSI history met NSSID criteria. In most studies more girls than boys met criteria (Table 1). The average age of onset for NSSI in those with NSSID ranged from 12.52 to 13.05 years (SD 1.73–3.53) [23, 50, 52]. The most common methods were cutting, banging/hitting, severe scratching, carving and scraping [23, 50, 53]. Several methods were reported, ranging from an average of 4.29–8 (SD 2.18–2.78) methods [23, 46, 50–53]. The functions most often endorsed by those who met NSSID criteria were affect regulation, self-punishment and anti-dissociation/feeling-generation [23, 46, 50, 53]. In clinical studies of adolescents and young adults with NSSID, 69.2–83.3% [50, 51] reported having made a suicide attempt, and in one study 24.4% reported having done so during the last month [23]. Among community adolescents who met criteria for NSSID, 20% reported that at least one of their self-injuries during the last year was a suicide attempt [46]. Several of those with NSSID in clinical and community samples with recurrent NSSI also had concurrent axis I diagnoses [23, 45, 50, 51, 53]. Mood disorders commonly co-occurred, with examples of 72.5% [53] and 79.5% [50] for depression. Anxiety disorders were also commonly reported (72.5–89%) [23, 51, 53], as was posttraumatic stress disorder (PTSD) with rates of 25.0–28.2% [50, 53]. In two studies of clinical adolescents with NSSID, 51.7% [23] and 20.5% [50] met criteria for BPD. High levels of emotional dysregulation [23, 53], low quality of life [52] and impairment [45, 52] have also been found in those meeting criteria for NSSID.

### DSM-5 NSSI criteria

#### Criterion A

In a self-injuring sample of inpatient and intensive outpatient adolescents and young adults, 85.5% endorsed criterion A, i.e., at least 5 days [52]. Rates of 76–77% were found in an outpatient clinical sample and also in a community sample of repetitive NSSI [51, 53], whilst a considerably lower endorsement of criterion A (20.8%) was found in a self-injuring adult community sample [55]. Of those who met NSSID criteria, 73.7% had performed NSSI ≥ 11 times during the last year and 26.3% had done so 5–10 times. More girls than boys had performed NSSI ≥ five times in this study of community adolescents [46]. Lengel and Mullins-Sweatt [42] asked 119 clinicians and NSSI experts to rate whether the NSSID criteria represented prototypic cases/symptoms of a self-injuring patient and 85% considered that five instances was prototypic. Absence of suicidal intent was endorsed as prototypic by 90%.

#### Criterion B

In one community study of adolescents [46], almost all (99.5%) of those with NSSID reported having engaged in NSSI with the expectation of relieving an interpersonal difficulty or negative feeling, or of inducing a positive feeling. A similarly high endorsement (87.2–87.7%) was found in inpatient adolescents with NSSID [50, 52]. Engaging in NSSI for a purpose was also thought to be a prototypical symptom by 71.9% of clinicians and NSSI experts [42]. In one study [53] 79% of young adults with NSSI met criterion B, compared to 66.4% in an adult community sample of self-injurers [55]. The earlier B criterion (current DSM-5 equivalent of B and C) was met by 97% of self-injuring outpatient adolescents and young adults [51]. Empirical studies that used the final DSM-5 [13] criteria and presented data for each subcriterion found B1 (relief) to be the most common [52, 55]. In adolescents, B3 (positive feeling) was least commonly endorsed [52]. Criterion B2 (to relieve interpersonal problems) was more often endorsed in a clinical sample including adolescents [52] than in an adult community sample [55]. In the study by Washburn and colleagues [52] patients rarely met criterion B without also meeting criterion C. Criterion B was further found to be associated with interpersonal functions of NSSI [53]. Girls reported expectations of relief from negative feelings and thoughts more often than boys [47].

#### Criterion C

Criterion C1 (interpersonal/psychological precipitant) was consistently met by nearly all participants. Of adolescents with NSSID, 97.4–100% endorsed criterion C1 [46, 50, 52]. In the study by Washburn and colleagues [52] there was an additionally high endorsement of criteria C2 (preoccupation) and C3 (urge). Of those who did not meet criteria for NSSID, very few failed to meet criterion C. Criterion C1 was also significantly associated with psychopathology and impairment [52]. Of those with self-injury, 81–98% [23, 51–53] met criterion C and 82.4% of self-injuring community adults met criterion C1 [55]. Psychological precipitants were more commonly reported in girls [46, 47]. Negative emotions/thoughts prior to NSSI was considered a prototypic symptom by 87.5% of clinicians, while frequent urge and preoccupation to engage in NSSI was relatively less so [42]. Similarly, preoccupation was reported by less than 50% of the adolescents with NSSID in the study by In-Albon and colleagues [50], while frequent urge was endorsed by 89.7%.

#### Criterion D

In a study of young adults [53] 91% of self-injurers met criterion D, which refers to behaviors that are not socially sanctioned. Eighty-eight percent of clinicians and NSSI experts thought this to be a prototypic symptom [42].

#### Criterion E

In one study of clinical self-injuring adolescents and young adults, 43% failed to meet NSSID criteria because they did not fulfill the distress or interference criterion [51]. The interviewers considered this criterion difficult to assess, since patients tended to report that their self-harm was helpful rather than distressing or impairing. In self-injuring samples, 41–64% met criterion E [51, 53]. In adolescents with NSSID, 76.8% [46] and 69.2% [50] reported that their NSSI caused them distress. However, a question whether adolescents desired help for their NSSI received a 79.5% endorsement [50]. In Andover’s [55] adult sample, 8.8% of self-injurers endorsed interferences in functioning, while 60.8% wanted to stop engaging in NSSI. The most common interferences reported were in academic and social (school) life [47], interpersonal relationships and schooling [46] and also leisure time [50]. More girls than boys acknowledged distress/impairment [46]. Criterion E had less than 50% endorsement as a prototypic symptom [42]. In a study of young adults, clinical characteristics such as emotion dysregulation, BPD, symptoms of depression, anxiety and stress were most strongly associated with criterion E, as were intrapersonal functions, and this criterion best distinguished those with NSSID from those with NSSI without NSSID [53].

#### Criterion F

In a self-injuring sample of young adults, 80% met exclusion criterion F [53], as did 98.2% of adolescents [52]. Several of the studies using self-report measures did not assess this criterion directly.

### NSSI disorder versus NSSI, clinical controls and borderline personality disorder

#### NSSI disorder versus NSSI

Compared to those with NSSI not meeting NSSID criteria, those with NSSID reported higher levels of psychopathology and significantly more interference in functioning [52, 53, 55], as well as more variety of NSSI methods [51–53] (Table 2). The NSSID group endorsed significantly higher levels of automatic functions (emotion relief, feeling generation) than the non-NSSID group [46, 53, 55], with average rates of automatic negative reinforcement of 2.43 (0.84) vs. 1.54 (0.81) and automatic positive reinforcement 2.08 (0.71) vs. 1.33 (0.51) in inpatient adolescents [50]; significantly higher levels of emotion dysregulation, 109.42 (21.79) vs. 94.26 (23.07) [53]; significantly higher levels of symptoms of depression, 18.68 (11.28) vs. 13.99 (9.86) indicating moderate vs. mild/moderate symptoms; anxiety symptoms, 15.12 (9.81) vs. 9.31 (7.23) indicating severe vs. mild symptoms and stress, 20.65 (10.00) vs. 14.20 (8.04) indicating moderate vs. mild symptoms in young adults with recurrent NSSI [53]. There were also significantly higher levels of symptoms of depression, anxiety, anger, posttraumatic stress and dissociation in community adolescents with NSSID compared to those with NSSI not meeting NSSID criteria [44] and significantly more smoking and drug use [46]. Significantly more community adolescents with NSSID reported experiences of adversities and maltreatment than adolescents with NSSI not meeting NSSID criteria [44], for example, bullying, 62.4 vs. 40.0%; emotional abuse, 77.4 vs. 40.8%; physical abuse from an adult within the family, 38.7 vs. 16.0% and sexual abuse, 36.6 vs. 8.4% [44]. Suicide ideation, 1.40 (1.17) vs. 1.08 (1.18), was also significantly higher in inpatient adolescents with NSSID compared to those with NSSI not meeting full criteria [52]. Concerning concurrent axis I diagnoses, significantly more young adults with NSSID had PTSD, 25.0 vs. 10.4%; BPD, 45.0 vs. 19.4%; bipolar disorder, 20.0 vs. 6.0%; social anxiety disorder, 37.5 vs. 19.4% and alcohol dependence, 40.0 vs. 17.9%, compared to individuals with recurrent NSSI not meeting NSSID criteria [53]. Among inpatient adolescents with NSSID there were significantly higher levels of BPD traits, 37.79 (11.35) vs. 33.38 (10.92) [52]. Importantly, the association between NSSID and psychopathology in the study by Gratz and colleagues [53] remained significant when controlling for BPD.

**Table 2:** Group differences when comparing NSSID vs. NSSI; NSSID vs. clinical controls; NSSI vs. BPD

References	Sample and age group	Groups being compared (*n*)	Variables	Results
Andover [55]	Community adults	NSSID (14) NSSI (111)	NSSI characteristics Impairment/functioning NSSI functions	NSSID > NSSI Those with NSSID reported NSSI on significantly more days in the past year, 86.64 (134.47) vs. 6.38 (35.89); that NSSI interfered significantly more with functioning, 28.6 vs. 6.3% and significantly higher levels of automatic functions, 3.79 (1.67) vs. 1.81 (1.88); 5.00 (2.00) vs. 2.41 (2.51)
Bracken-Minor and McDevitt-Murphy [54]	College young adults	NSSID+/BPD+ (29) NSSID+/BPD− (33) NSSI−/BPD+ (37)	Demographics NSSI methods NSSI functions Emotion dysregulation Distress tolerance	NSSID+/BPD+ > NSSID+/BPD− Those with BPD reported significantly higher levels of emotion dysregulation, 105.28 (22.95) vs. 88.31 (21.56); self-punishment, 3.90 (2.04) vs. 2.39 (2.12); anti-suicide 2.41 (2.16) vs. 1.06 (1.87) and anti-dissociation, 2.38 (1.86) vs. 1.42 (1.73) functions and significantly more individuals reported cutting, 82.8 vs. 30.3% and burning, 48.3 vs. 24.2%
Glenn and Klonsky [23]	Clinical inpatient and partial hospitalization adolescents	NSSID (98) CC with and without NSSI (100)	Demographics Diagnoses Suicidal thoughts and behaviors Emotion dysregulation Loneliness	NSSID > CC^d^ Significantly more individuals with NSSID were female, 86.7 vs. 61%; had an anxiety disorder, 73.5 vs. 41.2%; mood disorder, 66.3 vs. 33.3%; bulimia, 18.3 vs. 0%; BPD, 51.7 vs. 14.9%; suicide ideation, 67.1 vs. 29.2%; suicide attempt, 24.4 vs. 8.6%; more total axis I disorders, 4.23 (2.52) vs. 2.35 (1.76); and significantly higher levels of emotion dysregulation, 117.94 (28.07) vs. 86.62 (29.94) and loneliness, 27.12 (6.66) vs. 22.29 (6.15)
Gratz et al. [53]	Community with recurrent NSSI young adults	NSSID (40) NSSI (67)	Demographics NSSI characteristics NSSI functions Emotion dysregulation BPD pathology Psychiatric diagnoses	NSSID > NSSI^e^ Significantly more individuals with NSSID met criteria for BPD, 45.0 vs. 19.4%; bipolar disorder, 20.0 vs. 6.0%; PTSD, 25.0 vs. 10.4%; social anxiety disorder, 37.5 vs. 19.4%; alcohol dependence, 40.0 vs. 17.9%; lifetime substance use disorder 65.0 vs. 37.0% and used burning as NSSI method, 55 vs. 31%; used a significantly greater number of NSSI methods, 6.45 (2.78) vs. 5.14 (2.69); significantly more overall interference and impairment associated with NSSI; significantly higher levels of emotional relief, 3.37 (0.96) vs. 2.73 (0.93) and feeling generation, 3.02 (1.20) vs. 2.24 (1.12) functions; emotion dysregulation, 109.42 (21.79) vs. 94.26 (23.07); symptoms of depression, 18.68 (11.28) vs. 13.99 (9.86); anxiety, 15.12 (9.81) vs. 9.31 (7.23); stress, 20.65 (10.00) vs. 14.20 (8.04) and BPD pathology 76.71 (13.20) vs. 67.89 (11.63)
In-Albon et al. [50]	Clinical inpatient adolescents	NSSID (41) NSSI without distress/impairment (12) CC (20)	Demographics NSSI characteristics NSSI functions Diagnostic correlates Clinical correlates Suicide attempts Smoking	NSSID > CC Significantly more individuals with NSSID had major depression, 79.5 vs 30.0%; relatively more individuals with NSSID had PTSD, 28.2 vs. 5%; suicide attempts, 69.2 vs. 20%; significantly higher symptoms of depression, 13.82 (4.56) vs. 8.84 (5.73); 36.32 (12.32) vs. 23.36 (13.11); emotion dysregulation, 123.42 (25.80) vs. 97.79 (24.14); externalizing, 21.31 (11.32) vs. 12.91 (1.74) and internalizing, 33.75 (10.04) vs. 25.28 (9.67) symptoms; borderline symptoms, 186.62 (64.93) vs. 120.47 (76.01) and lower GAF scores, 53.70 (10.17) vs. 59.55 (6.40) NSSID > NSSI Individuals with NSSID had significantly higher levels of automatic functions of NSSI, 2.43 (0.84) vs. 1.54 (0.81); 2.08 (0.71) vs. 1.33 (0.51)
Manca et al. [14]	School adolescents	NSSID (30) NSSI (24;34;111)^c^	R-NSSI-Q tendencies	NSSID > NSSI Individuals with NSSID had significantly higher R-NSSI-Q scores
Odelius and Ramklint [51]	Outpatient clinical adolescents and young adults	NSSID (18) NSSI (21)	NSSI frequency Psychiatric diagnoses Suicide ideation, attempts and risk	NSSID > NSSI Those with NSSID had a significantly higher mean number of self-harm methods, 8 vs. 6, and significantly more had a high suicide risk, 50 vs. 29%
Selby et al. [45]^a^	Clinical outpatient adults	NSSID+/BPD− (65) BPD+/NSSI∓ (24) CC (482)	Demographics Psychiatric diagnosis Global functioning Psychopathology	BPD > NSSID, CC Significantly more females, 88 vs. 51 vs. 52% and experience of abuse, 54 vs. 28 vs. 16%, in individuals with BPD BPD, NSSID > CC Significantly more individuals with BPD and NSSID had a depressive disorder, 46 vs. 42 vs. 25%; experience of abuse, 54 vs. 28 vs. 16%; mood swings, 96 vs. 80 vs. 40%; recurrent conflicts with others, 54 vs. 49 vs. 16%; strange beliefs or thoughts, 63 vs. 49 vs. 23%; aggression, 50 vs. 31 vs. 13% compared to clinical controls. Those with BPD and NSSID also had more previous treatment, 3.6 (1.4) vs. 2.9 (1.6) vs. 2.3 (1.6); higher clinical global impression¬, 4.5 (1.0) vs. 4.4 (1.2) vs. 3.4 (1.4); lower GAF scores, 56.8 (13.5) vs. 53.7 (13.3) vs. 64.0 (11.3); higher levels of depressive symptoms, 22.2 (10.2) vs. 24.8 (12.9) vs. 14.3 (10.6); suicide ideation, 6.4 (8.0) vs. 9.2 (11.7) vs. 1.9 (4.1); suicide attempts, 0.92 (0.86) vs. 0.74 (0.86) vs. 0.17 (0.44); less time since most recent suicide attempt, 3.9 (1.2) vs. 3.6 (1.4) vs. 4.8 (0.60) NSSID > CC Significantly more individuals with NSSID had mood disorders (25 vs. 10% for dysthymia; 11 vs. 2% for bipolar disorder); cluster A PDs, 6 vs. 0.1% and higher levels of anxiety symptoms, 22.8 (15.2) vs. 14.2 (11.9)
Washburn et al. [52]	Clinical inpatient, partial hospitalization intensive outpatient adolescents and young adults	NSSID (378) NSSI (133)	Demographics NSSI characteristics Psychopathology Quality of life Functional impairment	NSSID > NSSI Those with NSSID reported significantly more frequent NSSI, 88.72 (104.80) vs. 42.91 (88.31); methods, 4.29 (2.78) vs. 3.21 (3.40); urge, 21.06 (7.86) vs. 16.83 (8.62); higher levels of psychopathology, 2.05 (0.63) vs. 1.74 (0.70); suicide ideation, 1.40 (1.17) vs. 1.08 (1.18); BPD traits, 37.79 (11.35) vs. 33.38 (10.92); higher impairment, 18.03 (9.86) vs. 15.34 (9.79) and lower quality of life, 49.22 (17.79) vs. 55.29 (18.74)
Ward et al. [43]^a^	Clinical outpatient adults	NSSID+/BPD− (65) BPD+/NSSI∓ (24) CC (482)	Treatment variables Clinical impairment at intake and termination Response to treatment	NSSID, BPD > CC Significantly more individuals with NSSID and BPD ended therapy prematurely, 64 vs. 64 vs. 49% and had lower levels of functioning at termination, 3.6 (1.5) vs. 4.2 (1.7) vs. 2.8 (1.4)¬; 63.8 (16.1) vs. 60.5 (15.3) vs. 70.0 (12.8) NSSID > BPD, CC Those with NSSID showed more improvement following therapy with higher global functioning scores at termination compared to intake NSSID > CC Those with NSSID showed a larger decrease on ratings of severity of illness at termination compared to intake
Zetterqvist et al. [46]^b^	School adolescents	NSSID (205) NSSI (883)	Demographics Functions of NSSI NSSI characteristics	NSSID > NSSI Significantly more individuals with NSSID were female, 83.4 vs. 49.9%; lived with only one parent, 32.4 vs. 21.0%; alone or at institution, 13.2 vs. 5.2%; had parents that were unemployed or on long-term sick leave, 26.1 vs. 12.5%; perceived some or serious financial difficulties in the family, 40.9 vs. 20.0%; reported past or present smoking, 72.5 vs. 51.6%; drug use, 24.9 vs. 12.3%; were enrolled in a vocational program, 54.6 vs. 49.7% or individual educational program, 9.8 vs. 5.3%. All functions of NSSI were endorsed by a higher proportion of adolescents with NSSID, especially the automatic functions
Zetterqvist et al. [44]^b^	School adolescents	NSSID (186) NSSI (630)	Maltreatment/adversities Trauma symptoms	NSSID > NSSI Significantly more adolescents with NSSID reported bullying, 62.4 vs. 40.0%; emotional abuse 77.4 vs. 40.8%; physical abuse 38.7 vs. 16.0%; sexual abuse, 36.6 vs. 8.4%; parental chronic adversity 69.4 vs. 53.5% and significantly higher levels of symptoms of depression, 12.71 (5.66) vs. 5.15 (4.44); anxiety, 9.35 (5.91) vs. 5.32 (4.35); anger, 9.35 (5.91) vs. 5.32 (4.35); posttraumatic stress, 14.72 (6.06) vs. 7.53 (5.65) and dissociation, 12.45 (6.29) vs. 6.60 (5.09)

*BPD* borderline personality disorder, *CC* clinical controls, *GAF* global assessment of functioning, *NSSI* nonsuicidal self-injury, *NSSID* nonsuicidal self-injury disorder, *PD* personality disorders, *PTSD* posttraumatic stress disorder, *R-NSSI-Q* Repetitive Non-Suicidal Self-Injury Questionnaire, ¬higher score indicates more serious illness.

^a^Same study sample.

^b^Same study sample.

^c^Intent uncertain group excluded due to not being applicable in the present fifth version of the Diagnostic and Statistical Manual of Mental Disorders.

^d^Over and above BPD.

^e^Over and above BPD except for depressive symptoms, PTSD and social anxiety disorder.

#### NSSI disorder versus clinical controls

Significantly more inpatient adolescents with NSSID reported suicide ideation, 67.1 vs. 29.2% and suicide attempts, 24.4 vs. 8.6% [23], compared to clinical adolescents. Furthermore, significantly more inpatient adolescents among those who met NSSID criteria had major depression, 79.5 vs. 30.0% [50]; anxiety disorder, 73.5 vs. 41.2%; mood disorder, 66.3 vs. 33.3%; bulimia, 18.3 vs. 0%; BPD, 51.7 vs. 14.9%; a higher total number of axis I diagnoses, 4.23 (2.52) vs. 2.35 (1.76) and reported loneliness compared to clinical controls [23]. Adolescents with NSSID also had significantly more internalizing and externalizing symptoms [50]; higher levels of emotion dysregulation and general psychopathology and impairment than clinical controls [23, 50]. The association between NSSID and clinical impairment in the study by Glenn and Klonsky [23] remained significant when controlling for BPD. An adult NSSID group also had significantly more general psychopathology and impairment [43, 45]; more symptoms of anxiety and depression [45]; more suicide attempts and ideation; were more often victims of abuse; had more previous treatment [45], ended therapy prematurely, had worse prognostic outcome after therapy than an axis I clinical comparison group but showed larger decreases on ratings of severity of illness from intake to termination as well as more improvement following therapy [43] (Table 2).

#### NSSI disorder versus borderline personality disorder

One study on adults distinguished potential NSSID from BPD. There were no differences in comorbidity and functional impairment between the groups. The BPD group, however, contained more women, 88 vs. 51% and reported higher rates of abuse, 54 vs. 28% [45]. The same sample was also used in a later study by Ward et al. [43], where those with NSSID showed greater improvement after treatment compared to intake than those with BPD. In one study [50] 80% of adolescents who met NSSID criteria did not meet criteria for BPD. Glenn and Klonsky [23] found that NSSID occurred independent of BPD. There was a significant overlap between NSSID and BPD, but the diagnostic overlap between BPD and other disorders was similar to that between BPD and NSSID. Odelius and Ramklint [51] also found that patients with NSSID had several comorbid diagnoses which were not concomitant with BPD. Bracken-Minor and McDevitt-Murphy [54] compared BPD-positive and BPD-negative self-injuring young adults and found preliminary support for a distinction, where those with BPD reported higher levels of emotion dysregulation, 105.28 (22.95) vs. 88.31 (21.56) and functions of self-punishment, 3.90 (2.04) vs. 2.39 (2.12); anti-suicide, 2.41 (2.16) vs. 1.06 (1.87) and anti-dissociation, 2.38 (1.86) vs. 1.42 (1.73). Furthermore, the NSSI methods cutting and burning were more often reported compared to those without BPD (Table 2).

### Assessment of NSSI disorder

Several studies have assessed NSSID criteria indirectly with instruments not originally developed for this purpose. The Clinician Administered Nonsuicidal Self-Injury Disorder Index (CANDI) [53] and the self-report measure The Alexian Brothers Assessment of Self-Injury (ABASI) [52] were designed to assess and identify NSSID. The CANDI showed good interrater reliability. The overall diagnostic agreement was 92%. There was a 100% agreement for criteria A, B, C, D and F and 92% for criterion E. Furthermore, internal consistency was adequate and there was support for construct validity. There was support for a two-factor solution on the ABASI, with all items assessing criterion B and criterion C loading on respective factor. Internal consistency was adequate. Item-total correlations showed that the ABASI item for criterion B3 was weakly correlated with the NSSI severity score. Test–retest reliability was moderate for the NSSID, good for criterion A and criterion C, but poor for criterion B. Test–retest was good for ABASI NSSI severity scores and moderate for criterion B and criterion C subscales. In-Albon and colleagues [50] constructed a clinical interview from the DSM-5 criteria which showed very good interrater reliability. Fischer et al. [49] used a German version of the Self-Injurious Thoughts and Behaviors Interview (SITBI) [56] to identify NSSID and found moderate agreement in test–retest and very good interrater reliability. They argued that NSSI may have been triggered in their sample by the inpatient clinical setting, hence influencing test–retest results. Fischer et al. [49] suggested extending SITBI to include items on functional impairment and distress to optimally match NSSID criteria.

## Discussion

Empirical data are now emerging on the DSM-5 [13] NSSID concerning prevalence rates, characteristics, proposed criteria, clinical correlates and independence from other disorders, which are important aspects when validating a new diagnosis [57]. Comparisons and conclusions are however limited by the fact that different versions of the criteria have been used and that not all criteria have been assessed or have been assessed indirectly [30]. In addition, the total number of empirical studies is still small, especially for those presenting the full final DSM-5 criteria, indicating that this is an area in need of further study. In view of the fact that limited reliability prevented the inclusion of an NSSI diagnosis in DSM-5 [40, 41], studies with psychometric data from instruments with structural assessment of NSSID [52, 53] have shown promising results.

### NSSI disorder criteria

Since NSSI has shown to be a common phenomenon in adolescents, both in clinical and community samples [2, 3], it is important to differentiate between those who engage in the behavior once or twice and those who do so more repetitively. In a sample of young adolescents with high endorsement of NSSI, for example, Bjärehed et al. [58] found that a high proportion of adolescents only reported low levels of frequent NSSI and also low levels of associated psychological problems. Previous research has shown support for a distinction between occasional and repetitive NSSI, with frequent NSSI being associated with more psychopathology [14, 58]. In several studies five instances has come to represent repetitive NSSI [14, 58]. With regard to the DSM-5 [13] cut-off of five instances, a study by Zetterqvist et al. [46] showed that a majority of adolescents in a community sample reported engaging in NSSI more than 11 times during the past year. In clinical child and adolescent psychiatry practice, adolescents often report far higher frequencies, giving the impression that five is perhaps a low limit for adolescents. This is thus an area that needs looking into in more detail. Furthermore, as criterion A is currently stated, no significance is given to potential differences between severe and minor NSSI methods in relation to the number of instances, and this also needs some further elaboration [30]. Some of the self-report measures used to operationalize NSSI criteria include NSSI methods where there might be uncertainty whether they induce actual bleeding, bruising or pain. As Washburn and colleagues [52] pointed out, this might result in an overestimation of criterion A. To address this, some studies have excluded some methods so as to arrive at conservative estimates [46, 52]. Most participants with NSSID, however, endorsed several different NSSI methods, which might reduce this risk. That NSSI was preceded by negative feelings or relational difficulties (C1) and relieved negative states (B1) were commonly endorsed criteria [23, 46, 50, 52]. Lengel and Mullins-Sweatt [42] also found that these features were assessed by many clinicians as prototypic symptoms of the NSSID diagnosis. Criteria B2, B3 and C2, C3 were relatively less frequently endorsed. Specifically, experiencing negative emotions prior to NSSI was highly endorsed, confirming the motivation for affect regulation as a central aspect of the NSSID construct. There was a clear difference between adults and adolescents in the endorsement of criterion B2 (resolving an interpersonal difficulty). This is in line with previous research showing that interpersonal functions are more common in adolescents than in adults [59, 60]. In one adolescent sample [52], criterion B3 (inducing positive feeling) was least commonly endorsed, and there is an ongoing discussion of the positive and negative aspect of the automatic reinforcement of NSSI [61–63]. Based on their results, Washburn and colleagues [52] raised the issue that perhaps criterion B is superfluous in relation to criterion C and that a combination of the two would result in more parsimonious criteria. In one study of adults, over 10% responded “I don’t know” to criterion B items [55]. Perhaps precipitating events are easier to consciously observe than consequences of behaviors. This could also imply that the wording of the B criterion needs to be clarified for a more precise definition. Can B3 also refer to pain, stimulation and satisfaction [62]? Selby et al. [30] have also pointed out that the B3 criterion could preferably be expanded to include feeling generation/anti-dissociation when feeling numb or empty [46, 59, 61, 62].

One potential explanation why more girls than boys meet NSSID criteria is perhaps that boys traditionally are less inclined to acknowledge the emotional and motivational aspects of the diagnosis [46, 47]. Interpretations of gender differences should, however, be made with caution since there was female overrepresentation in samples. Several of the empirical studies in this review have drawn attention to the fact that criterion E received a relatively lower endorsement. That NSSI tends to be regarded as a solution, reducing distress rather than causing it, has previously been problematized by Wilkinson and Goodyer [33] with regard to the wording of criterion E. Clinicians also rated criterion E as less prototypic, suggesting that while clinicians were concerned with NSSI and its consequences, individuals with NSSI may not always perceive themselves as impaired in their everyday lives [42]. It is somewhat problematic that different operationalizations of criterion E have been used in the empirical studies of NSSID. Some, for example, have assumed impairment based on the fact that participants are in psychiatric inpatient clinics, while others have asked if participants wanted help for their NSSI. Compared to other diagnoses, such as ADHD or depression, where the distress/impairment criterion is more easily applied, it is perhaps necessary with further instructions how this criterion should be operationalized so as not to exclude individuals incorrectly. Gratz et al. [53] showed that criterion E best distinguished NSSID from those with NSSI not meeting criteria for the disorder, which implies that it is important for the validity of the construct and, as such, potentially functions appropriately by screening out those without distressing or impairing NSSI.

### NSSI disorder as a separate diagnostic entity

Using the DSM-5 criteria [13], a sample of individuals was identified who had more general psychopathology and impairment than both clinical controls and those with NSSI not meeting criteria for NSSID, preliminarily supporting that NSSID can be reliably identified among self-injurers. Importantly, the differences remained significant after BPD was controlled for [23, 53] and NSSID was preliminary found to be distinguishable from BPD [50, 54]. In adolescents, for example, each disorder explained unique variance in emotion regulation deficits [23]. Furthermore, BPD-positive self-injurers with NSSID reported higher levels of emotion dysregulation than BPD-negative self-injurers with NSSID [54]. Support for the independence of NSSID should be based on an overlap between NSSID and BPD to the same extent as other disorders, as pointed out by Glenn and Klonsky [23]. Similarly, suicidal behaviors also co-occur with depression, PTSD, substance abuse and eating disorders, for example, as well as several other clinical behaviors and thus an overlap between NSSI and suicidal behaviors is not necessarily evidence per se against a distinction between the two.

### Future work

Future work in the research field of NSSI would benefit from a unified conceptualization of NSSI with standardized assessment measures in order to facilitate comparisons and achieve more consistent results. The proposed NSSID diagnostic criteria [13] are a step towards a mutually agreed-upon conceptualization [3]. Although most criteria were possible to apply and were assessed as prototypical, some clarification of criteria is perhaps needed in order to facilitate clinical assessment. Future studies are needed to assess whether all suggested criteria are equally meaningful clinically. The prevalence rates of the final DSM-5 [13] NSSID criteria need to be further verified in both clinical and community groups of adolescents by other methods than self-report, such as diagnostic interviews, to further assess reliability and validity of a potential NSSID diagnosis. It is also important to collect more data on male samples. Further studies on overlapping and unique correlates to NSSID are also needed, as are longitudinal studies in order to examine risk factors and the prognosis of NSSID, and its relationship to diagnostic neighbors and suicidal behaviors over time.

## Conclusion

When the DSM-5 NSSID criteria were used in the reviewed empirical studies, a group of adolescents and young adults was identified that was clinically more severe in comparison both with those with NSSI not meeting NSSID criteria and with clinical controls. There was also preliminary support for the independence of NSSID and a distinction in relation to BPD. In order to accumulate data to validate and reliably assess a potential NSSID, further empirical studies are needed using the full and final DSM-5 [13] criteria.
